# Pragmatic Two‐Stage Design for Clinical Trials With Treatment Selection

**DOI:** 10.1002/sim.70683

**Published:** 2026-07-27

**Authors:** Paul Horton, Yajun Mei

**Affiliations:** ^1^ H. Milton Stewart School of Industrial and Systems Engineering Georgia Institute of Technology Atlanta GA USA; ^2^ Department of Biostatistics, School of Global Public Health New York University New York NY USA

**Keywords:** clinical trial design, treatment selection, two‐stage design

## Abstract

Clinical trials are inherently complex and become more challenging when selecting one treatment from many while also evaluating the efficacy. In this paper, we develop a pragmatic design that balances sample efficiency and practicality through a two‐stage sequential approach. The first stage involves adaptive arm selection and early termination, while the second stage optimizes sample allocation. Inspired by the Kiefer‐Weiss problem, the proposed method provides an efficient solution to an optimization framework that minimizes a weighted sum of the expected number of patients, probability of selecting a bad arm, and the probability of a global Type II error subject to constraints on the Family‐Wise Error Rate and maximum sample size. The framework employs stochastic optimization to find the design parameters for the global minimum. We compare the performance against fixed and multi‐arm multi‐stage designs over a range of test conditions with varying error costs and treatment‐response models. Our method improves the cost and practicality of a clinical trial for medical treatments or pharmaceutical development.

## 1 Introduction

Selecting the optimal treatment from several alternatives in a clinical trial is important to maximize both the therapeutic impact and the likelihood of regulatory approval. Traditionally, treatment selection has included an initial evaluation of efficacy and safety with more rigorous evaluations in later phases. Improved decision making for finding the optimal treatment has typically come at the cost of additional samples, leading to a higher cost, or adopting a sequential trial design. Although sequential trial designs can improve efficiency, they introduce complexities in planning and operations, particularly due to the large variability in sample size requirements between the trial's minimum and maximum limits. Furthermore, there is growing pressure within clinical research to reduce the number of participants exposed to less effective or suboptimal treatments, adding to the complexity of the trial design. As a result, there is a need for pragmatic methods which are financially efficient by minimizing the expected number of patients, utilize open‐source software, limit operational challenges and maintain compliance with regulatory standards.

Inspired by the Kiefer‐Weiss [1] problem, we propose the Pragmatic Two‐Stage Design (P2SD) for clinical trials that balances the efficiency and operational challenges of a sequential design while also incorporating adaptive features for improved performance. We propose a new framework for evaluation that is based on the minimization of a weighted sum of the expected number of patients, the probability of making a bad selection, and the probability of global Type II error under constraints on the Family‐Wise Error Rate (FWER) and maximum sample size. An important feature of the proposed method is the decoupling of the decision about the hypotheses from the selection of the best arm. In this way, the trial can proceed to a second stage without the placebo if the hypothesis is rejected in the first stage. Similarly, all treatment arms excluding the best can be dropped in the first stage, while the second stage remains for making a decision on the null hypothesis. This added flexibility makes the proposed method adaptive in multiple senses and allows for explicit control over the probability of making a good selection. With appropriate parameter selection, the proposed method can become a seamless two‐stage adaptive trial with treatment selection in the first stage and efficacy confirmation in the first or second stage.

The remainder of this paper is organized as follows. We first provide a background on the problem and review the relevant literature in Section 2. In Section 3, we describe the problem formulation and introduce the relevant notation. Section 4 outlines the decision‐making process of the proposed method, detailing how the decision variables are optimized. Finally, Section 5 presents the results for sample efficiency as well as a comparison of the practicality of relevant methods. We conclude with a discussion of contributions and potential extensions in Section 6.

## 2 Background

Below, we broadly review concepts relevant to the scope of this paper. First, we generally describe the parts of a clinical trial design in Section 2.1. We then discuss other treatment selection methods and their differences in Section 2.2.

### 2.1 Clinical Trial Design

The size and scope of a clinical trial largely depend on the stage of development for the treatment. In the case of the pharmaceutical industry, the FDA [2] categorizes drug development into Phase I, II, and III with progressively larger scopes and more participants for later phases.

Phase II trials commonly select a single treatment out of a candidate group to be evaluated in later stages, as is commonly the case for dose finding trials with a shared control arm [3]. Treatment selection trials often evaluate fewer than five alternatives to limit the cost and complexity as is often the case with dose finding trials in the pharmaceutical industry [4, 5, 6]. While these tend to have fewer overall observations than Phase III trials, the implications of the decisions made during this trial influence the probability of success in Phase III and therapeutic impact in the market. As the P2SD method is designed to efficiently select the best treatment, Phase II trials are the primary applications and the focus of this paper.

### 2.2 Treatment Selection

There are multiple ways to construct a clinical trial to find the best treatment from several alternatives. In a fixed design, there is a pre‐determined number of patients for the trial. In many cases, this is managed through a single stage trial where all observations are collected prior to the analysis. A recent method for fixed designs, known as Drop‐the‐Losers (DTL), uses sequential testing with a pre‐specified number of arms dropped at each stage [7]. This semi‐fixed design combines the characteristics of dropping the worst performing arms with the practical value of a fixed number of total patients. Thall et al. [8] use a two‐stage design where only a single treatment can continue to the second stage regardless of the probability of making a good selection. Many treatment selection algorithms using fully sequential decision making are based on the “fixed confidence multi‐armed bandit” problem such as the Thompson sampling method for a fully sequential setting where sampling is proportional to a posterior probability of an arm being the best [9]. Fully sequential designs are most prevalent in Phase I trials where the responses are quicker to measure. Other sequential methods include group‐sequential and multi‐arm multi‐stage trials (MAMS) which proposed a flexible decision to drop under‐performing arms or stop the trial entirely when a single arm rejects the null hypothesis [10]. The MAMS formulation has become widely adopted as in [11]. Under this setting, equal sample allocation has been shown to be most powerful when the variance is equal among arms [12]. For more information on group sequential designs, see [13].

Adaptive designs have recently been applied to treatment selection problems. Broadly, adaptive designs can respond to dynamic estimates of unknown parameters, such as the treatment effect size. Applications include sample size recalculation [14], response adaptive randomization [15], and adding or dropping arms [11]. Yuan et al. [16] propose a design with a fixed number of samples for the second stage but allocate a new sample into an arm at an adaptive probability. Other adaptive designs allocate new observations to an arm depending on the response from previous observations [17]. Zheng and Chow [18] consider a two‐stage design for dose‐finding within a seamless adaptive design without explicit control of the Type I and II errors. The additional flexibility of adaptive designs creates an opportunity for improved sample efficiency but this complexity also creates challenges for explicit control over error rates.

There are also multiple ways to consider what would be a good decision for the treatment selection. One criterion is to evaluate whether the selection was the best arm such as chosen by Stallard and Todd [19]. Another criterion for evaluating the treatment selection is to consider whether it was ϵ‐good as evaluated in [20] and proposed more generally by Tamhane and Bechhofer [21]. In this formulation, ϵ is a pre‐specified parameter such that any arm within ϵ of the best performing arm would be considered a good selection. As in both Stallard and Todd [19], and Kelly et al. [20], the evaluation of each arm is done with a comparison to a placebo which means the test statistics become dependent. In addition to using a different method for solving, there are two key differences in our setup and the previous papers: the structure of an optimization problem and the constraint on the maximum number of samples. The method from [19] does not reference any possibility of stopping collection on the placebo group if the best performing arm proves superior while continuing to sample to improve the probability of selecting the best.

## 3 Problem Formulation

Suppose a researcher has several candidate treatments and needs to select one to proceed with for further evaluation. There are K candidates which will be evaluated against a placebo. We assume that the target response follows an independent normal distribution. For each group, the data are given by:

$$\begin{array}{ll} X_{01},X_{02},\ldots \overset{\text{iid}}{∼}𝒩(\mu_{0},\sigma^{2}) & \\ X_{11},X_{12},\ldots \overset{\text{iid}}{∼}𝒩(\mu_{1},\sigma^{2}) & \\ \vdots & \\ X_{K1},X_{K2},\ldots \overset{\text{iid}}{∼}𝒩(\mu_{K},\sigma^{2}) & \end{array}$$

where the parameters of interest are $\mu_{k}$ for k=0,…,K with $\mu_{0}$ indicating the mean for the control group. For each $X_{ki}$, k denotes the treatment group and i indicates the sample number. To highlight our main ideas, we assume that the common variance $\sigma^{2}$ is known. The methods can be adapted for unknown variances by changing the distributions of test statistics from Normal distributions to t‐distributions. We assume that there is a reasonably large total number of subjects in the first stage for variance estimation, for example, $(K+1)(\tau_{1}-1)\geq 30$ if $\tau_{1}$ is the sample size per arm in the first stage. See more specific discussions in Section 6. We use the notation that $\mu_{\left[1\right]}\leq \cdots \leq \mu_{\left[K\right]}$ for the ranking of the true means. The treatment arm with the highest mean is denoted by $K^{*}=\{k|\mu_{k}=\mu_{\left[K\right]}\}$. We use μ as the vector of true mean values such that $\mu =(\mu_{0},\mu_{1},\ldots ,\mu_{K})$.

We consider a set of K hypothesis tests where the mean for each treatment is compared against the control:

$$\begin{array}{ll} H_{0}^{\left(1\right)}:\mu_{1}-\mu_{0}\leq 0\;\text{against}\;H_{1}^{\left(1\right)}:\mu_{1}-\mu_{0}>\Delta . & \\ \vdots & \\ H_{0}^{\left(K\right)}:\mu_{K}-\mu_{0}\leq 0\;\text{against}\;H_{1}^{\left(K\right)}:\mu_{K}-\mu_{0}>\Delta . & \end{array}$$

In each alternative hypothesis above, we use Δ as our threshold to denote the minimum clinically relevant effect. Treatment selection is a primary component of this problem so a successful outcome would be to find that at least one of the treatments has the minimum required effect. We can therefore use the Union‐Intersection Test (UIT) construction:

$$H_{0}:\mu_{1}-\mu_{0}\leq 0,\mu_{2}-\mu_{0}\leq 0,\ldots ,\;\text{and}\;\mu_{K}-\mu_{0}\leq 0$$

against

$$H_{1}:\mu_{1}-\mu_{0}\geq \Delta ,\;\text{or}\;\mu_{2}-\mu_{0}\geq \Delta ,\;\ldots ,\;\text{or}\;\mu_{K}-\mu_{0}\geq \Delta$$

In the above construction, all local null hypotheses must be true to accept the global null while at least one of the local null hypotheses must be rejected to the reject the global null. As a result, we define the sets $\Theta_{0}$ and $\Theta_{1}$ as:

$$\begin{array}{ll} \Theta_{0} & =\{\mu =(\mu_{0},\ldots ,\mu_{K})|\mu_{k}-\mu_{0}\leq 0\;\text{for all}\;k\},\\ \Theta_{1} & =\{\mu =(\mu_{0},\ldots ,\mu_{K})|\mu_{k}-\mu_{0}\geq \Delta \;\text{for at least one}\;k\} \end{array}$$

Simply, $\Theta_{0}$ is the set of all possible μ for which the null hypothesis is true while $\Theta_{1}$ is the set for which the alternative hypothesis is true where at least one treatment arm has a clinically significant effect.

In this article, we follow the well‐known Kiefer‐Weiss problem [1] where the true μ might come from a third space, $\Theta^{*}$, that might be different from the hypotheses being tested. In other words, we define the third space $\Theta^{*}$ as

$$\begin{array}{l} \Theta^{*}=\{\mu =(\mu_{0},\ldots ,\mu_{K})|0\leq \underset{1\leq k\leq K}{\max}(\mu_{k}-\mu_{0})\leq \Delta \}. \end{array}$$

At a high‐level, when testing $\Theta_{0}$ against $\Theta_{1},$ it is often the most challenging to make a decision when the true μ actually comes from $\Theta^{*}$, or more specifically, when $\mu_{k}-\mu_{0}=\Delta /2$. By minimizing the expected sample size under a pre‐specified $\mu \in \Theta^{*}$, the Kiefer‐Weiss problem provides a useful mathematical formulation that is able to control the maximum expected sample size. See [22] and [23] for more discussions and applications in group sequential designs for clinical trials.

We describe our general two‐stage design with decision variables $\left(J,\hat{K},\delta \right)$, where J is the set of arms proceeding to the second stage, $\hat{K}$ is the selected treatment, and δ is the decision on the null hypothesis where δ=0 indicates that we accept $H_{0}$ and δ=1 indicates we reject it. When selecting the treatment arm, we aim to control the probability of our selection being in a set of true means within a specified distance to the maximum given by $\hat{K}\in L$ where $L=\{k|\mu_{k}\geq \mu_{\left[K\right]}-\zeta \}$ and ζ is a pre‐specified, non‐negative constant. In the case where $0\leq \zeta <\mu_{\left[K\right]}-\mu_{\left[K-1\right]}$, we have simply $L=\{K^{*}\}$. The set of treatments, L, is potentially distinct from the set of effective treatments. For example, the trial may correctly select the best treatment even when no treatments are effective.

Given our goal of a pragmatic approach, we follow researchers such as [24] and [8] who recognize a two‐stage trial design offers a practical solution by reducing the complexity of planning and operations while achieving improved sample efficiency through sequential analysis. Generally, clinical trials are viewed as a cost so we seek a design that will minimize the risk associated with a trial as a function of three components shown in (1). First, we have the expected number of samples $E_{\mu^{*}}[(K+1)\tau_{1}+|J|\tau_{2}]$ where J is the set of arms which continue to the second stage, $\tau_{1}$ and $\tau_{2}$ are the sample size per arm at the respective stage, and the vector of means, $\mu^{*}$, is pre‐specified and can be either estimated from an earlier analysis or come from an expected response curve as described by Bretz et al. [25]. Second, there is the probability of making a bad selection under $\mu^{*}$ given by $P_{\mu^{*}}(\hat{K}\notin L)$. Finally, we include a component for the probability of incorrectly accepting the null hypothesis when the alternative is true. We consider the worst case for this probability in the constraint which is upper bound by the error rate $\tilde{\beta }$. To align with FDA regulations, we include a constraint on the FWER that is set to a level of α. We use $\sup_{\mu \in \Theta_{0}}P_{\mu }(\delta =1)$ to denote the FWER which is the maximum probability of incorrectly rejecting $H_{0}$. Additionally, there is a constraint on the maximum number of samples which serves as a proxy for financial restrictions on the trial. We note that there is a cost associated with recruiting additional participants to a clinical trial. As such, a trial sponsor, such as a pharmaceutical company, may not want to find the most effective treatment at any cost for this stage of development given the potential to fail. Simply, we want to minimize the expected number of patients and weighted costs of incorrect decisions with constraints on the FWER and maximum number of samples.

$\underline{\text{Optimization Problem}:}$ Given a $\mu^{*}\in \Theta^{*}$ along with its treatment set $L=\{k|\mu_{k}\geq \mu_{\left[K\right]}-\zeta \}$, find the two‐stage trial design with decision variables $\left(J,\hat{K},\delta \right)$ and the stage‐wise sample sizes $\left(m_{1},m_{2}\right)$ that minimize the risk

(1) $$E_{\mu^{*}}\left[(K+1)\tau_{1}+|J|\tau_{2}\right]+\lambda_{1}P_{\mu^{*}}(\hat{K}\notin L)+\lambda_{2}\tilde{\beta }$$

subject to the constraints

(2) $$\begin{array}{cc} & \sup_{\mu \in \Theta_{0}}P_{\mu }(\delta =1)\leq \alpha ,\\ & \sup_{\mu \in \Theta_{1}}P_{\mu }(\delta =0)\leq \tilde{\beta },\\ & m_{1}+m_{2}\leq N_{max},\\ & \tau_{1}=\lfloor m_{1}/(K+1)\rfloor \\ & \tau_{2}=\left\{\begin{array}{ll} \lfloor m_{2}/|J|\rfloor \; & \text{if}\;J\neq ø\\ 0\; & \text{if}\;J=ø \end{array}\right.\\ & m_{1},m_{2},\tau_{1},\tau_{2}\in {\mathbb{Z}}^{+} \end{array}$$

where $\lambda_{1},\lambda_{2}\in [0,\infty )$ are weights assigned to making erroneous decisions, and $N_{max}\in {\mathbb{Z}}^{+}$ is a positive integer specifying the maximum number of patients for the entire trial. We can consider $\tilde{\beta }=\inf\{\beta |\sup_{\mu \in \Theta_{1}}P_{\mu }(\delta =0)\leq \beta \}$. This is not a pre‐specified value and thus it has the Type II error rate under the worst configuration given $\mu \in \Theta_{1}$.

For this optimization problem, we consider a special class of designs that are specified by the stage‐wise sample size parameters $\left(m_{1},m_{2}\right)$ and thresholds $\left(u_{1},\ell_{1},u_{2},h\right)$. These six parameters completely determine the decisions $\left(J,\hat{K},\delta \right)$ which are random variables depending on the observed data.

When the variance $\sigma^{2}$ is unknown, we would assume that $N_{\max}\geq K+31,$ and then add an additional constraint $(K+1)(\tau_{1}-1)\geq 30$, so that we have enough data to estimate $\sigma^{2}$ well at the end of the first stage.

For a detailed table of notation, see Table E1 in Appendix E.

## 4 Proposed Method

In this section, we define the P2SD method for solving (1) subject to the constraints in (2) through the design parameters $\left(m_{1},m_{2},h,u_{1},u_{2},\ell_{1}\right)$ where $m_{1}$, $m_{2}$ are the sample sizes allocated the respective stage, h∈[0,∞) is a distance that controls which treatments are selected to continue to the second stage, and $u_{1},u_{2},\ell_{1}$ are the decision boundary parameters for the respective stage. In Section 4.1, we first describe the algorithm to make a selection on the best arm, $\hat{K}$, and then the decision to reject or accept the null hypothesis. In Section 4.2, we describe the process for optimization.

### 4.1 Decision Rules

**FIGURE 1:**
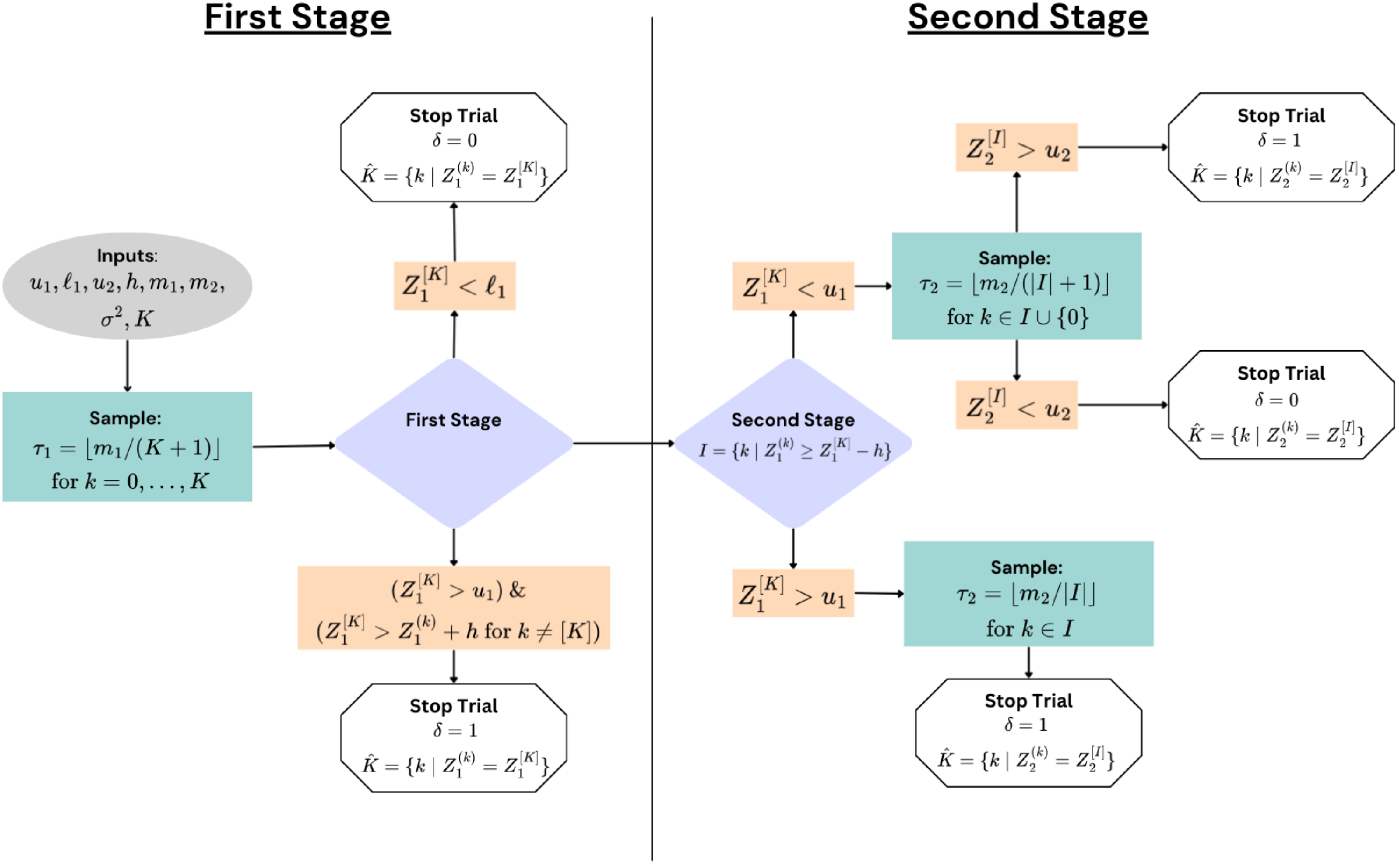
Flowchart for visualizing the decision process for the two P2SD method with sampling operations in aqua, decision criteria in orange, and stopping nodes in white.

Our proposed two‐stage procedure combines two fixed‐sample tests: we sample a fixed number, $\tau_{1}$, per arm in the first stage. At the end of the first stage, we select the set of arms, J, to continue to the second stage. In the second stage, take $\tau_{2}$ samples per arm that adapts to the number of arms which continue to the second stage. We treat this as a sequential problem so the test statistic at stage j=1,2 for treatment arm 1≤k≤K is:

(3) $$Z_{j}^{\left(k\right)}=\left(\frac{\sum_{i=1}^{N_{j}}X_{ki}}{N_{j}}-\frac{\sum_{i=1}^{N_{j}}X_{0i}}{N_{j}}\right)/\sqrt{\frac{2\sigma^{2}}{N_{j}}}$$

which is the standardized cumulative difference in means. For reference, $Z_{j}^{\left(k\right)}∼𝒩(0,1)$ under the global null hypothesis when μ=0. Similar to the notation for the means, we use $Z_{j}^{\left[1\right]}\leq \cdots \leq Z_{j}^{\left[K\right]}$ for ordering the test statistics. We use $N_{j}$ as the cumulative number of samples through the jth stage for each arm. With the assumption of equal variance between all groups, we maintain an equal distribution of samples between all active trial arms. See Figure 1 for further details. Below, we explain the process for decisions in each stage based on the trial outcomes:

Stage 1: The control arm and all K treatment arms are active so we define $\tau_{1}=\lfloor m_{1}/(K+1)\rfloor$ for the number of samples for each arm, where ⌊.⌋ denotes the floor function (rounded down to the nearest integer). There are three potential outcomes at the end of the first stage:
  a. If $Z_{1}^{\left[K\right]}<\ell_{1}$, we stop the trial and accept the null hypothesis that the best treatment arm does not have a clinically significant effect. In this case, we declare that $\hat{K}=\{k|Z_{1}^{\left(k\right)}=Z_{1}^{\left[K\right]}\}$ and J={ø}.
  b. If $Z_{1}^{\left[K\right]}>u_{1}$ and $Z_{1}^{\left[K\right]}>Z_{1}^{\left[K-1\right]}+h,$ we stop the trial and accept the alternative hypothesis. In this case, we declare that $\hat{K}=\{k|Z_{1}^{\left(k\right)}=Z_{1}^{\left[K\right]}\}$ is the best treatment arm with minimum required effect and J=ø.
  c. If $\ell_{1}\leq Z_{1}^{\left[K\right]}\leq u_{1}$, or if $Z_{1}^{\left[K\right]}>u_{1}$ but $Z_{1}^{\left[K\right]}\leq Z_{1}^{\left[K-1\right]}+h,$ we continue to the second stage.
Stage 2: If the trial continues to the second stage, we proceed with any treatment arm k in *the active treatment set*
(4) $$\begin{array}{l} I=\{k\in \{1,\ldots ,K\}|Z_{1}^{\left(k\right)}\geq Z_{1}^{\left[K\right]}-h\}. \end{array}$$
  Note that |I|≥1, since at least the arm with the maximum $Z_{1}^{\left[K\right]}$ belongs to I. A subtlety in the second stage is whether to continue sampling the control arm k=0 which depends on the value of $Z_{1}^{\left[K\right]}$ at the end of the first stage. There are two cases:
  a. If $\ell_{1}\leq Z_{1}^{\left[K\right]}\leq u_{1}$, then we sample the control arm k=0 in addition to the active treatment set I in (4). In other words, the active sampling set J=I∪{k=0}.
  b. If $Z_{1}^{\left[K\right]}>u_{1}$ but $Z_{1}^{\left[K\right]}\leq Z_{1}^{\left[K-1\right]}+h,$ then we do not sample the control arm. In this case, the active sampling set is J=I in (4).
  In either case, we set the number of samples for each active arm as $\tau_{2}=\lfloor m_{2}/|J|\rfloor$ if J≠ø and $\tau_{2}=0$ if J=ø. At the end of the second stage, we use Z2[I]=maxk∈I Z2(k) as the maximum test statistic for treatments in set I, and declare that $\hat{K}=\{k|Z_{2}^{\left(k\right)}=Z_{2}^{\left[I\right]}\}$ which picks the treatment arm with the largest test statistic in the second stage. With a two‐stage sequential design, we set $u_{2}=\ell_{2}$ to ensure a decision at the last stage.

**FIGURE A1:**
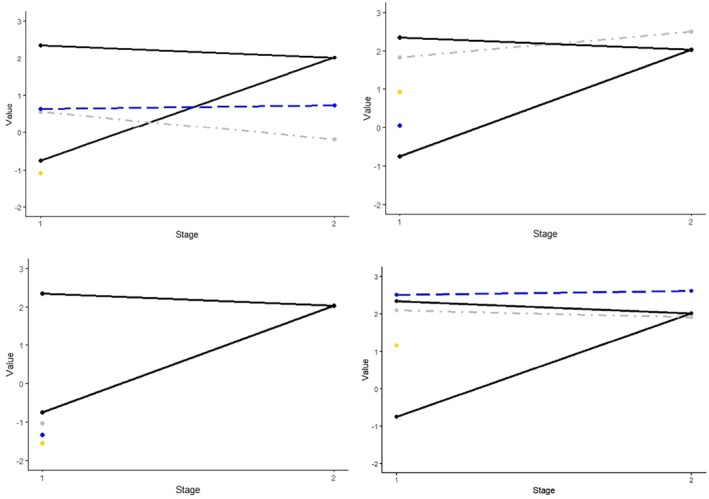
Visualization of examples shown in Table 1 where the decision boundaries are in black (solid), Treatment 1 in grey (dot‐dash), Treatment 2 in blue (long dash) and Treatment 3 in gold (dot).

We include Table 1 with four examples to further demonstrate the proposed decision process. In the first example, two treatments proceed to the second stage with no decision to accept or reject. In the second stage, Treatment 2 was selected and the decision was to accept $H_{0}$. In the second example, Treatment 1 was selected in the first round so it was the only arm that proceeded to the second stage where $H_{0}$ was rejected. The third example has $H_{0}$ accepted in the first round with Treatment 1 selected and the test stops. The final example rejects $H_{0}$ in the first round and selects Treatment 2 in the second round. These examples are shown visually in Figure A1 in Appendix A.

**TABLE 1:** Example decisions when $u_{1}=2.34$, $\ell_{1}=-0.75$, $u_{2}=2.02$, and h=0.82. The value in bold indicates which arm was selected as the best.

	Stage	Treatment 1	Treatment 2	Treatment 3
Example 1	$Z_{1}^{\left(k\right)}$	0.56	0.64	−1.08
	$Z_{2}^{\left(k\right)}$	−0.19	0.74	—
Example 2	$Z_{1}^{\left(k\right)}$	1.83	0.06	0.92
	$Z_{2}^{\left(k\right)}$	2.49	—	—
Example 3	$Z_{1}^{\left(k\right)}$	−1.03	−1.34	−1.55
	$Z_{2}^{\left(k\right)}$	—	—	—
Example 4	$Z_{1}^{\left(k\right)}$	2.10	2.51	1.15
	$Z_{2}^{\left(k\right)}$	1.91	2.61	—

To clarify the relationship between the optimization function (1), constraints (2), and decision variables, we include further details on the construction of possible events. We use the two‐stage nature of our proposed method to split each component into two parts based on the stage. For example, the expected number of patients for the entire trial can be split up by evaluating events in the two stages:

(5) $$\begin{array}{cc} & {𝔼}_{\mu^{*}}[(K+1)\tau_{1}+|J|\tau_{2}]={𝔼}_{\mu^{*}}[\text{Samples in Round 1}]\\ & \;+{𝔼}_{\mu^{*}}[\text{Samples in Round 2, continue in Round 1}]. \end{array}$$

Furthermore, we know that the number of samples in the first round is fixed based on the number of arms in the trial and the samples per arm in the first stage. The number of samples in the second stage is a random variable as the trial may stop in the first stage due to efficacy or futility:

(6) $$\begin{array}{ll} & {𝔼}_{\mu^{*}}\left[(K+1)\tau_{1}+|J|\tau_{2}\right]={𝔼}_{\mu^{*}}\left[(K+1)\times \tau_{1}\right]\\ & \;+{𝔼}_{\mu^{*}}\left[\overset{K}{\underset{k=1}{\sum }}I\left(k\in I\cup \{0\};\;\ell_{1}\leq Z_{1}^{\left[K\right]}\leq u_{1}\right)\times \tau_{2}\right]\\ & \;+{𝔼}_{\mu^{*}}\left[\overset{K}{\underset{k=1}{\sum }}I\left(k\in I;\;u_{1}<Z_{1}^{\left[K\right]}\leq Z_{1}^{\left[K-1\right]}+h\right)\times \tau_{2}\right], \end{array}$$

where I(·) is the indicator function and I is the active treatment set in (4).

Below, we consider the connection of the constraint $\sup_{\mu \in \Theta_{0}}P_{\mu }(\delta =1)\leq \alpha$ to the decision variables as a summation of probabilities to reject the null hypothesis in either stage. To control the FWER, we must ensure that the maximum probability for any condition under the null hypothesis is less than α. For our procedure, the Type I error probability is monotonically increasing over each $\mu_{k}$ when $\mu =(\mu_{1},\ldots ,\mu_{K})\in \Theta_{0}$, and thus it is maximized when all the treatments have no effect such that $\mu_{0}=\mu_{1}=\cdots =\mu_{K}=0$ which we denote as μ=0. When we evaluate over the two stages, the formula for the upper limit on the FWER becomes:

(7) $$P_{\mu =0}(\delta =1)=P_{\mu =0}(\delta_{1}=1)+P_{\mu =0}(\delta_{2}=1,\delta_{1}=ND),$$

where $\delta_{1}$ denotes a decision in the first stage where $\delta_{1}=ND$ indicates no decision on the null hypothesis. In the first stage, all the treatment arms are active so we reject $H_{0}$ only if this value exceeds the upper threshold from the first stage. The decision is based off the maximum test statistic so we can rewrite this as the union of the events that any of the arms exceed the upper boundary:

(8) $$P_{\mu =0}(\delta_{1}=1)=P_{\mu =0}(Z_{1}^{\left[K\right]}\geq u_{1})=P_{\mu =0}\left({⋃}_{k=1}Z_{1}^{\left(k\right)}\geq u_{1}\right).$$

The P2SD method is a sequential design so the decision in the second stage depends on the results from the first stage. In this case, there must be no decision in the first stage and the maximum test statistic for any of the arms that continued must exceed the upper boundary in the second stage:

(9) $$P_{\mu =0}(\delta_{2}=1,\delta_{1}=ND)=P_{\mu =0}(Z_{2}^{\left[I\right]}\geq u_{2},Z_{1}^{\left[K\right]}\geq \ell_{1},Z_{1}^{\left[K\right]}\leq u_{1})$$

The global Type II error rate is maximized when there is only one treatment arm with a significant effect while the others are insignificant which can be expressed as $\mu_{1}=\Delta$ and $\mu_{0}=\mu_{2}=\cdots =\mu_{K}=0$. We will refer to this as the Least‐Favorable‐Configuration (LFC). We will use the following notation to simplify the expression $\sup_{\mu \in \Theta_{1}}P_{\mu }(\delta =0)=P_{LFC}(\delta =0)$.

(10) $$\tilde{\beta }=P_{LFC}(\delta_{1}=0)+P_{LFC}(\delta_{2}=0,\delta_{1}=ND)$$

Similar to the calculation for the FWER, we accept $H_{0}$ in the first stage if the maximum test statistic is less than the lower boundary. Acceptance in the second stage requires no decision in the first and the maximum test statistic of the arms which continued to be below the boundary in the second stage.

To make a good selection for the treatment arm, the selection must be in the set L which contains all arms within a pre‐specified distance, ζ, from the arm with the largest test statistic:

$$\begin{array}{cc} & P_{\mu^{*}}(\hat{K}\in L)=P_{\mu^{*}}(\text{Good selection in stage I})\\ & \;+P_{\mu^{*}}(\text{Good selection in stage II, no selection in stage I}). \end{array}$$

Selecting a treatment in the first stage, denoted by ${\hat{K}}_{1}$, requires that either the maximum test statistic be at least a distance, h, from all the others or the trial stops entirely with the maximum test statistic less than the lower boundary in the first stage:

(11) $$\begin{array}{cc} P_{\mu^{*}}({\hat{K}}_{1}\in L) & =\underset{k\in L}{\sum }P_{\mu^{*}}\left(\left({⋂}_{l=1}^{K}Z_{1}^{\left(k\right)}\geq Z_{1}^{\left(l\right)}+h\right)\cup \right.\\ & \left.\left({⋂}_{l=1}^{K}Z_{1}^{\left(k\right)}<\ell_{1}{,⋂}_{l=1}^{K}Z_{1}^{\left(k\right)}\geq Z_{1}^{\left(l\right)}\right)\right). \end{array}$$

Making a good selection in the second stage requires no selection in the first stage in addition to adapting to the random number of arms which proceed to the second stage. Thus, our formulation for the probability of making a good selection is the second stage is:

(12) $$\begin{array}{cc} & P_{\mu^{*}}({\hat{K}}_{2}\in L,{\hat{K}}_{1}=NS)=\underset{k\in L}{\sum }P_{\mu^{*}}\\ & \;\left({⋂}_{l\in I}(Z_{2}^{\left(k\right)}\geq Z_{2}^{\left(l\right)}),Z_{1}^{\left[K\right]}\geq \ell_{1},Z_{1}^{\left(k\right)}\geq Z_{1}^{\left[K\right]}-h,|I|>1\right), \end{array}$$

where ${\hat{K}}_{1}=NS$ indicates there was no treatment selection in the first stage.

### 4.2 Optimization

In the previous section, we outlined the decision process for a given set of inputs. Below, we describe our proposed method for finding the optimal values for the design parameters $\left(m_{1},m_{2},h,u_{1},u_{2},\ell_{1}\right)$.

First, we show how the components of the optimization problem can be modeled as multivariate normal random variables. Under the null hypothesis, the maximum probability of a Type I error occurs when μ=0. For this, all the test statistics have the same distribution so we can define the maximum probability of rejecting the null hypothesis in the first stage as:

(13) $$\begin{array}{cc} & P_{\mu =0}(Z_{1}^{\left[K\right]}\geq u_{1})=P_{\mu =0}\left({⋃}_{k=1}^{K}(Z_{1}^{\left(k\right)}=Z_{1}^{\left[K\right]},Z_{1}^{\left(k\right)}\geq u_{1})\right)\\ & \;=K\times P_{\mu =0}\left(Z_{1}^{\left(1\right)}\geq u_{1},{⋂}_{k\neq 1}Z_{1}^{\left(1\right)}\geq Z_{1}^{\left(k\right)}\right). \end{array}$$

The above union of events depends on each arm being the maximum in the first stage which means the events are disjoint so the probability of the union is equivalent to the summation of the individual probabilities. The probability of a rejection in the second stage is more complex than the above equation and requires enumerating all K possible scenarios where $Z_{1}^{\left(k\right)}=Z_{1}^{\left[K\right]}\;\text{for}\;k=1,\ldots ,K$ along with all combinations of I and the |I| scenarios where $Z_{2}^{\left[I\right]}=Z_{2}^{\left(k\right)}\;\text{for}\;k\in I$. For other components which include a union of events, we employ the inclusion‐exclusion principle to convert to an intersection of events. We include an example in Appendix B for a single series of events.

For the above function, each component has limits to specify for the multivariate normal random variable. With $Z_{1}^{\left(1\right)}\geq u_{1}$, we have a lower bound of $u_{1}$ with an upper bound of ∞. For the components in the intersection, with $Z_{1}^{\left(1\right)}\geq Z_{1}^{\left(2\right)}$ as an example, we convert to $Z_{1}^{\left(1\right)}-Z_{1}^{\left(2\right)}\geq 0$ which has a lower bound of zero and an upper bound of ∞.

These events may share observations through the common control or have a temporal correlation from shared observations for each arm through the two stages. There is a need to understand the relationship to generate a covariance matrix to calculate a multivariate normal probability. Below, we characterize the correlations for test statistics:

$$\begin{array}{ll} Cov(Z_{j}^{\left(k\right)},Z_{l}^{\left(m\right)}) & =\left\{\begin{array}{ll} \sqrt{\frac{\tau_{1}}{\tau_{1}+\tau_{2}}}\; & k=m,\;j\neq l,\\ \frac{1}{2}\sqrt{\frac{\tau_{1}}{\tau_{1}+\tau_{2}}}\; & k\neq m,\;j\neq l,\\ \frac{1}{2}\; & k\neq m,\;j=l. \end{array}\right.\\ Cov(Z_{j}^{\left(k\right)},Z_{l}^{\left(m\right)}-Z_{l}^{\left(n\right)}) & =\left\{\begin{array}{ll} \frac{1}{2}\sqrt{\frac{\tau_{1}}{\tau_{1}+\tau_{2}}}\; & k=m,\;j\neq l,\\ -\;\frac{1}{2}\sqrt{\frac{\tau_{1}}{\tau_{1}+\tau_{2}}}\; & k=n,\;j\neq l,\\ \frac{1}{2}\; & k=m,\;j=l,\\ -\;\frac{1}{2}\; & k=n,\;j=l,\\ 0\; & \text{otherwise}. \end{array}\right. \end{array}$$

With the specified means, correlation matrices, upper and lower bounds for each event, we use the method from the mvtnorm package by [26] in R to calculate the multivariate normal random variable.

With an explicit function for both the optimization problem and constraints, it remains to select an algorithm to optimize. An optimization method that has theoretical properties guaranteeing global optimality would be ideal but the inherently discrete nature for the number of samples makes this a challenging mixed integer programming problem and inherently non‐convex. To our knowledge, even the relaxed version of this problem has not been evaluated for convexity so it is unclear what kind of guarantees any optimization algorithm might provide. With six design parameters ($m_{1},m_{2},h,u_{1},u_{2},\ell_{1}$), a grid search is infeasible. We note earlier in this section that there is a combinatorial aspect of this problem which is computationally challenging for algorithms which require many iterations. We instead pursue algorithms that offer good feasible solutions that exhibit consistent empirical convergence behavior. There are many stochastic optimization algorithms with relatively fast convergence that find a suitable feasible solution. Among those, we consider simulated annealing as a solution for this problem [27]. Briefly, simulated annealing is a Markov Chain Monte Carlo method that will transition to a new point when the solution is better but also has a non‐zero probability of transitioning to a worse solution. The probability to transition to a worse state is given by $\exp(-\frac{\left(f-f^{\prime }\right)}{T})$ where f is the candidate score, $f^{\prime }$ is the current score, and T is the temperature. The temperature has a decay function that reduces the probability of selecting a worse solution over time. The cooling rate for the temperature is an exponential decay given by $T=T_{0}r^{i}$ where $T_{0}$ is the initial temperature, r∈(0,1) is the decay factor, and i is the iteration. We implement a custom function for generating new candidates from a uniform distribution with a range that decays based on the iteration. When generating new candidates, the upper limit for the number of samples is set to align with $N_{max}$ to respect the constraint from (2).

To begin, we first convert optimization Problem (1) with constraints (2) to a penalized form:

(14) minm1,m2,hu1,ℓ1,u2𝔼μ∗[(K+1)τ1+|J|τ2]+λ1Pμ∗(K^∉L)+λ2β˜+λ3max(0,Pμ=0(δ=1)−α)

The values of $\lambda_{1}$, $\lambda_{2}$ are user‐specified to account for the cost of identifying an incorrect arm and mistakenly accepting $H_{0}$ while $\lambda_{3}$ is selected to be sufficiently large to restrict the search to the feasible range. The max(0,·) operator ensures that there is no penalty applied when the FWER is sufficiently low. The input, α, is user specified based on the regulatory requirements of the trial. We additionally relax the integer constraint for the number of observations for the optimization step. After solving, we set $\tau_{1}=\lfloor \tau_{1}\rfloor$ to re‐implement the integer constraint while also respecting the constraint on the maximum number of samples.

**FIGURE 2:**
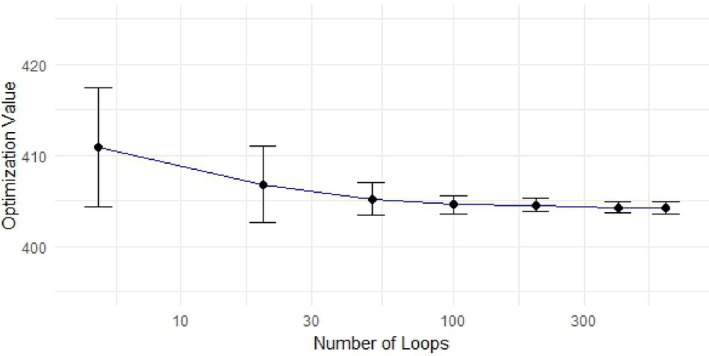
Parameter tuning for number of loops of simulated annealing with $\lambda_{1}=\lambda_{2}=500$, α=0.025 where error bars show the 95% confidence interval (±1.96σ).

Good convergence properties for optimization algorithms such as simulated annealing depend on suitable values of tuning parameters. Additionally, the stochastic nature of simulated annealing brings randomness to the returned solution emphasizing the need for evaluating convergence. Figure 2 evaluates the optimization value returned as a function of the number of inner loops (iterations) within simulated annealing. There is negligible improvement after 100 loops as the computational time scales linearly with this parameter. We find relatively small variance of the optimization value indicating we are successfully converging to a global optimum. Table C1 in the Appendix shows the increase in computation time associated with increasing the number of inner loops and Table C2 shows that the design parameters are relatively consistent between each solution after convergence.

## 5 Results and Analysis

In this section, we report numerical simulation results when evaluating the performance of the proposed P2SD method under various settings. Our numerical setup follows the configuration in [20] that selects the optimal treatment from K=3 treatment arms and considers a significant effect for any of the treatment arms to be Δ=0.249 with each arm having a variance $\sigma^{2}=0.249$. Since this configuration aims to find the best arm, this corresponds to our formulation by setting ζ=0 and $L=\{K^{*}\}$. Since our hypotheses are one‐sided (instead of two‐sided with the forms of $|\mu_{k}-\mu_{0}|\geq \Delta$), we decide to adopt a threshold of α=0.05/2=0.025 for the FWER, and a maximum sample size of $N_{\max}=500$ in our constraints in (2). This allows us to easily adopt the existing baseline methods for fair comparison.

In our numerical results below, we will consider a total of 10 distinct true $\mu \in \Theta^{*}\subset R^{K=3}$, depending on its maximum treatment effect and response curve (e.g., relative values of other components). In particular, two maximum values are considered: $\mu^{\left[K\right]}=\Delta$ or Δ/2. The former corresponds to the standard formulation of minimizing the expected sample size under the alternative hypothesis $\Theta_{1}$, and the latter highlights the Kiefer‐Weiss problem of minimizing the maximum expected sample sizes over $\mu^{*}\in \Theta^{*}$. In addition, for each of the two maximum values $\mu^{\left[K\right]}=\Delta$ or Δ/2, the following five different response curves, μ, from [25] are considered:

1. Linear: $\mu =\mu^{\left[K\right]}(1,2/3,1/3)$;
2. Least Favorable Configuration (LFC): $\mu =\mu^{\left[K\right]}(1,0,0)$;
3. Step: $\mu =\mu^{\left[K\right]}(1,2/3,2/3)$;
4. Logistic: $\mu =\mu^{\left[K\right]}(1,0.960,0.707)$;
5. Convex: $\mu =\mu^{\left[K\right]}(1,0.321,0)$.

**FIGURE D1:**
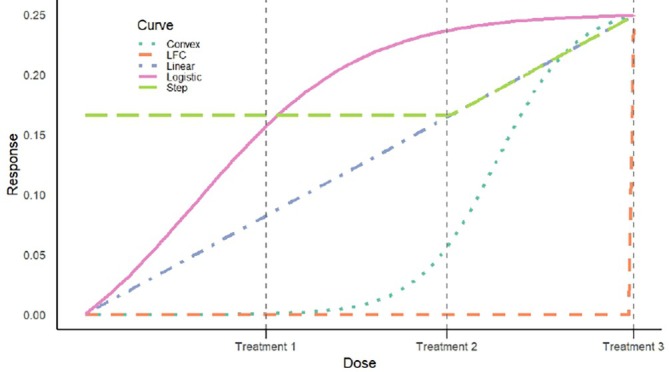
Visual of response curves evaluated in Section 5.

Figure D1 in Appendix D visualizes these five response curves. These 10 distinct true μ allow us to evaluate how well the methods perform on practical scenarios, as well as how robust our proposed P2SD method is.

In addition, all results below are relative to the penalized optimization problem in (14) with a pre‐specified pair $\left(\lambda_{1},\lambda_{2}\right)$ and a sufficiently large $\lambda_{3}$ that the original constraints in (2) are satisfied. We will first focus on the choices of $\lambda_{1}=\lambda_{2}=500$, for example, when the weights of making wrong decisions are comparable with the maximum sample size $N_{\max}=500,$ and we later discuss the effects of choosing other values of $\lambda_{1}$ and $\lambda_{2}.$ Results are compiled over 10 000 simulations with the lowest value for each configuration in bold.

We will compare our proposed P2SD method with 5 baseline designs: 1 fixed‐design, 1 two‐stage design, and 3 designs with three or more stages. For better presentation, we split our results into three subsections. In Subsection 5.1, we compare our methods with two simple baseline designs (i.e., single‐stage and two‐stage) under those 10 distinct true $\mu \in \Theta^{*}$ values. In Subsection 5.2, we compare our methods against three complex baseline designs with each having three or stages, not only under those 10 distinct true $\mu \in \Theta^{*}$, but also under different choices of $\left(\lambda_{1},\lambda_{2}\right)$. Finally, in Section 5.3, we quantify the operational and practical effects of each evaluated design and also evaluate the robustness of our proposed P2SD method with respect to misspecified μ values.

### 5.1 A Comparison With Two Simple Baselines

To compare the performance of our proposed P2SD method, in this subsection, we consider two simple baseline designs: one is a single‐stage fixed design, and the other is a two‐stage design from Magirr, Jaki, and Whitehead [10] abbreviated as MJW. Parameters for these two baseline methods are configured as:

- The single stage fixed design has 328 samples that rejects $H_{0}$ if $Z_{1}^{\left[K\right]}>2.33$.
- The MJW method uses J=2, β=0.1, and an equal distribution of samples between the first and second stages with boundaries $\ell_{j}=(0,2.355)$ and $u_{j}=(3.33,2.355)$.

With these parameters, these two baseline designs have a FWER of 0.025 when μ=(0,0,0), and a maximum sample size less than 500 which allows us to fairly compare all methods.

Table 2 compares our proposed P2SD method with these two baseline designs under 10 distinct true $\mu^{*}=\mu \in \Theta^{*}$ as stated in the previous subsection. The table includes each component of optimization (1) along with the optimization value which is referred to as the score. Compared to the fixed design, two‐stage designs, either MJW or our proposed P2SD, have improved scores and improved efficiency. Moreover, our proposed P2SD method has the minimum score among these three methods under each of these 10 different scenarios. This is consistent with our mathematical formulation in which our proposed P2SD method is designed to optimize the score function.

**TABLE 2:** Comparison against methods with two or less stages. Rows correspond to proposed and reference methods with the columns indicating the five response curves. The score indicates the value of optimization (1) with the value in bold indicating the best for that response curve. All methods have a FWER less than 0.025.

Setting	Method		#1 (Linear)	#2 (LFC)	#3 (Step)	#4 (Logistic)	#5 (Convex)
$\mu^{\left[K\right]}=\Delta$	**P2SD**	$E_{\mu }[(K+1)\tau_{1}+|J|\tau_{2}]$	278.0±0.8	254.4±0.8	287.8±0.8	261.4±0.8	263.0±0.8
		$P_{\mu }(\hat{K}\in L)$	0.846	0.991	0.760	0.479	0.974
		$P_{\mu }(\delta =1)$	0.881	0.885	0.902	0.928	0.871
		Score	**405**	**317**	**450**	**548**	**330**
	**Fixed**	$E_{\mu }[(K+1)\tau_{1}+|J|\tau_{2}]$	328	328	328	328	328
		$P_{\mu }(\hat{K}\in L)$	0.851	0.999	0.741	0.499	0.985
		$P_{\mu }(\delta =1)$	0.827	0.807	0.851	0.911	0.800
		Score	499	426	543	676	432
	**MJW**	$E_{\mu }[(K+1)\tau_{1}+|J|\tau_{2}]$	335.1±1.3	302.7±1.2	343.4±1.3	340.4±1.2	314.3±1.3
		$P_{\mu }(\hat{K}\in L)$	0.813	0.912	0.755	0.496	0.904
		$P_{\mu }(\delta =1)$	0.923	0.910	0.938	0.969	0.907
		Score	473	392	511	637	408
$\mu^{\left[K\right]}=\Delta /2$	**P2SD**	$E_{\mu }[(K+1)\tau_{1}+|J|\tau_{2}]$	247.0±0.5	256.2±0.6	252.2±0.5	236.7±0.5	266.5±0.5
		$P_{\mu }(\hat{K}\in L)$	0.593	0.845	0.618	0.400	0.784
		$P_{\mu }(\delta =1)$	0.273	0.258	0.279	0.342	0.255
		Score	533	404	518	620	439
	**Fixed**	$E_{\mu }[(K+1)\tau_{1}+|J|\tau_{2}]$	328	328	328	328	328
		$P_{\mu }(\hat{K}\in L)$	0.631	0.910	0.554	0.437	0.830
		$P_{\mu }(\delta =1)$	0.276	0.231	0.304	0.362	0.238
		Score	609	470	649	706	510
	**MJW**	$E_{\mu }[(K+1)\tau_{1}+|J|\tau_{2}]$	390.7±0.6	364.5±0.6	398.0±0.6	401.4±0.6	373.5±0.6
		$P_{\mu }(\hat{K}\in L)$	0.663	0.915	0.591	0.440	0.847
		$P_{\mu }(\delta =1)$	0.347	0.305	0.379	0.456	0.318
		Score	604	452	648	726	495

In particular, Table 2 highlights two key distinctions between the MJW and our proposed P2SD method. First, the MJW method makes a treatment selection in the first stage if the trial stops regardless of the difference in performance between the arms, whereas our proposed P2SD method drops non‐significant treatment arms in the first stage, thereby leading to an improved probability of making a correct treatment selection in the second stage. Second, the P2SD method has an improved relative efficiency to the MJW method which can be attributed to decoupling the decision on the hypothesis from the treatment selection. This results in early stopping of the control arm in cases with enough evidence. The price we need to pay is a slightly lower global power.

It is also interesting to compare the results of Table 2 between two scenarios of $\mu^{\left[K\right]}=\Delta$ and $\mu^{\left[K\right]}=\Delta /2$; the results for $\mu^{\left[K\right]}=\Delta /2$ have similar trends although the relative performance gap shrinks. In addition, all the methods have an increased probability of making incorrect selections due to the smaller effect sizes. These are consistent with our intuition or theoretical understanding.

### 5.2 A Comparison With Three Complex Baselines

In this subsection, we compare our proposed two‐stage P2SD method against more complex baseline designs with three or more stages. The purpose is to illustrate that our proposed method is comparable with these complex baselines that are supposed to be more efficient in the sense of smaller expected sample sizes. Of course, these baselines are designed for other purposes, and do not have explicit forms for optimizing according to our formulation in (1).

We will consider three baselines with each having three or more stages: (1) Kelly‐Stallard‐Todd (KST) in Kelly et al. [20]; (2) Drop‐the‐Losers (DTL) in Wason et al. [7]; and (3) Magirr‐Jaki‐Whitehead (MJW) in [10]. Since these baselines are not designed for optimizing (1), we must find parameters manually which adhere to the constraints on maximum sample size and FWER. Below, we define the parameters used for fair comparison:

- The KST method uses the 5 stage design from their paper with the decision boundary $\ell_{j}=(-3.17,3.07,9.02,14.43,17.00)$ and $u_{j}=(10.68,11.82,13.44,15.16,17.00)$ with ϵ=0.05.
- The DTL configuration has a 3:2:1 structure dropping one arm at each interim analysis, 40 samples per arm for each stage, and uses the threshold c=2.30 for rejection.
- The MJW method uses four stages, β=0.1, an O'Brien‐Flemming upper boundary, and a fixed lower boundary with parameters $\ell_{j}=(0,0,0,2.37)$ and $u_{j}=(4.74,3.35,2.73,2.37)$.

In particular, simulations confirmed that all these baseline methods had a FWER of 0.025 when μ=(0,0,0), and also have a maximum number of samples less than 500. Thus the comparisons are fair under the constraints in (2).

For completeness, we briefly explain how these baseline designs make decisions. The KST method will drop treatment arms if they are a distance, ϵ, from the maximum test statistic but the trial stops when the maximum crosses the decision boundary regardless of the amount of evidence for the best treatment. The DTL method drops a pre‐specified number of arms at each interim analysis and makes a decision on the hypothesis at the final stage. The MJW method evaluates each hypothesis individually, stops sampling a treatment arm if the test statistic crosses the lower boundary, and stops the trial completely if a test statistic crosses the upper boundary.

Table 3 compares our proposed P2SD method with these 3 more complex baselines under those 10 distinct true $\mu^{*}=\mu \in \Theta^{*}$ in the beginning of Section 5 when $\lambda_{1}=\lambda_{2}=500$. From this table, we can infer that our proposed P2SD design balances the decisions on arm selection and efficacy according to the penalties, $\lambda_{1}$ and $\lambda_{2}$, rather than prioritizing one component of the optimization function at the cost of the others. The results show that the P2SD is competitive against methods with more stages strictly based on the weighted average of expected samples and probability of making good decisions. Of course, we need to acknowledge that the tuning parameters for the baseline methods were not exhaustively optimized for problem (1) thus the comparison might be limited. There may be tuning parameters for each baseline method which can be further optimized to improve their performance.

**TABLE 3:** Comparison against methods with three or more stages. Rows correspond to proposed and reference methods with the columns indicating the five dose‐response curves. The score indicates the value of optimization (1) with the value in bold indicating the best for that dose‐response curve.

Setting	Method		#1 (Linear)	#2 (LFC)	#3 (Step)	#4 (Logistic)	#5 (Convex)
$\mu^{\left[K\right]}=\Delta$	**P2SD**	$E_{\mu }[(K+1)\tau_{1}+|J|\tau_{2}]$	278.0±0.8	254.4±0.8	287.8±0.8	261.4±0.8	263.0±0.8
		$P_{\mu }(\hat{K}\in L)$	0.846	0.991	0.760	0.479	0.974
		$P_{\mu }(\delta =1)$	0.881	0.885	0.902	0.928	0.871
		Score	**405**	317	**450**	**548**	**330**
	**KST** (ϵ=0.05)	$E_{\mu }[(K+1)\tau_{1}+|J|\tau_{2}]$	180.0±0.8	177.1±0.7	179.9±0.8	173.7±0.8	179.1±0.8
		$P_{\mu }(\hat{K}\in L)$	0.681	0.933	0.583	0.448	0.863
		$P_{\mu }(\delta =1)$	0.779	0.803	0.797	0.885	0.782
		Score	438	309	484	548	346
	**DTL** (3:2:1)	$E_{\mu }[(K+1)\tau_{1}+|J|\tau_{2}]$	360	360	360	360	360
		$P_{\mu }(\hat{K}\in L)$	0.839	0.997	0.741	0.502	0.979
		$P_{\mu }(\delta =1)$	0.935	0.938	0.934	0.969	0.933
		Score	471	392	521	640	401
	**MJW** ‐ 4 stages	$E_{\mu }[(K+1)\tau_{1}+|J|\tau_{2}]$	315.1±0.8	273.2±0.6	322.8±0.8	310.8±0.8	288.9±0.7
		$P_{\mu }(\hat{K}\in L)$	0.854	0.988	0.776	0.508	0.977
		$P_{\mu }(\delta =1)$	0.930	0.905	0.938	0.973	0.905
		Score	436	327	482	604	348
$\mu^{\left[K\right]}=\Delta /2$	**P2SD**	$E_{\mu }[(K+1)\tau_{1}+|J|\tau_{2}]$	247.0±0.5	256.2±0.6	252.2±0.5	236.7±0.5	266.5±0.5
		$P_{\mu }(\hat{K}\in L)$	0.593	0.845	0.618	0.400	0.784
		$P_{\mu }(\delta =1)$	0.273	0.258	0.279	0.342	0.255
		Score	533	404	518	620	439
	**KST** (ϵ=0.05)	$E_{\mu }[(K+1)\tau_{1}+|J|\tau_{2}]$	185.6±0.8	180.0±0.7	187.5±0.8	190.2±0.8	180.7±0.8
		$P_{\mu }(\hat{K}\in L)$	0.526	0.709	0.455	0.386	0.642
		$P_{\mu }(\delta =1)$	0.268	0.234	0.281	0.347	0.233
		Score	521	424	559	596	458
	**DTL** (3:2:1)	$E_{\mu }[(K+1)\tau_{1}+|J|\tau_{2}]$	360	360	360	360	360
		$P_{\mu }(\hat{K}\in L)$	0.633	0.885	0.550	0.431	0.809
		$P_{\mu }(\delta =1)$	0.378	0.343	0.400	0.480	0.347
		Score	575	449	616	676	487
	**MJW** ‐ 4 stages	$E_{\mu }[(K+1)\tau_{1}+|J|\tau_{2}]$	351.9±1.0	312.2±1.0	363.4±1.0	366.9±0.9	325.8±1.0
		$P_{\mu }(\hat{K}\in L)$	0.653	0.851	0.568	0.434	0.809
		$P_{\mu }(\delta =1)$	0.368	0.310	0.405	0.477	0.326
		Score	573	434	627	697	469

**FIGURE 3:**
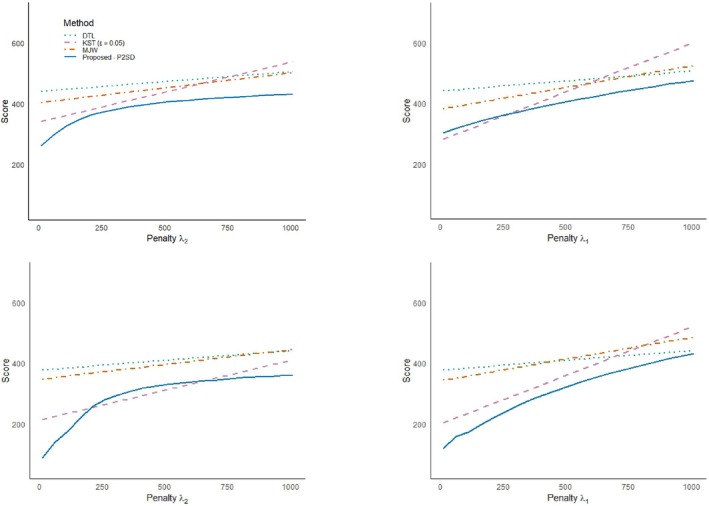
Method comparison when varying incorrect decision penalty with $\mu =\mu^{*}=\Delta (1,2/3,1/3)$ and $\sigma^{2}=0.249$. In clockwise order starting with the graph in the top left, $\lambda_{1}=500$, $\lambda_{2}=500$, $\lambda_{2}=100$, and $\lambda_{1}=100$.

Recall that $\lambda_{1}$ is the weight assigned for a bad arm selection while $\lambda_{2}$ penalizes for incorrectly accepting the null hypothesis. Thus it is useful to discuss the effects of different choices of tuning parameters $\lambda_{1}$ or $\lambda_{2}$. Figure 3 illustrates the optimization value of (1) for our proposed P2SD method and three baseline methods when varying either $\lambda_{1}$ or $\lambda_{2}$ with the other constant under the linear response curve μ=Δ(1,2/3,1/3) with Δ=0.249. Different clinical trials will have different relative costs for making bad decisions so this graph shows how well each method performs on a range of decision costs. Across all configurations, the P2SD method has either the best or second best performance. In cases when both penalties are small, such as in the bottom row towards the origin, the P2SD method optimizes towards a small number of samples due to the relatively small weight of incorrect decisions. It is worth noting that the largest relative benefits occur in settings when there is either a small or large penalty for making incorrect decisions on either the arm selection or the null hypothesis. This is understandable as the baseline methods require a priori specifying either the number of samples used or the global power rather than treating those as parameters to optimize. These graphs show the P2SD method can perform well with varying decision weights for specific applications.

### 5.3 Operational and Robustness Considerations

Since trial designs have effects beyond the financial cost of conducting the trial under the pre‐specified hypotheses, in this subsection, we compare different designs on operational and robust metrics that relate to the practicality of the design. Specifically, we consider the allocation of patients to ineffective treatments (placebo), difference between minimum and maximum total number of samples and per treatment arm, and the number of interim analyses. Moreover, we also investigate the robustness of our proposed P2SD method that was designed for a pre‐specified $\mu^{*}$ when the true underlying parameter vector $\mu \neq \mu^{*}$.

In Table 4, we compare the distribution of samples in total and between arms under the linear response curve when $\lambda_{1}=\lambda_{2}=500$ for the methods evaluated in Section 5.2. On average, the proposed method allocates relatively fewer observations to the placebo, 28.6%, compared to the KST and DTL methods at 35.9% and 33.3%, respectively. This is ethically important as patients subjected to ineffective placebos could otherwise receive benefit from one of the treatments. The relatively larger share of observations allocated to the treatment arms provides additional power for correctly determining the arm with the highest mean. Additionally, the P2SD method is the only method which allocates the highest percentage of observations to the best treatment arm. This is a result of decoupling the decision about the hypothesis from the treatment selection allowing early stopping of the control arm. The proposed method has a smaller difference in both the min/max number of samples per arm and total compared to the KST and MJW methods. Specifically, the KST method could have as few as 100 samples or as many as 500 in increments of 50. Contrast that with the proposed method having either 180 or 330 samples which reduces the operational and planning complexity. Additionally, there is at most a single interim analysis for the proposed method compared to potentially four, two, and three interim analyses for the KST, DTL, and MJW methods, respectively. Fewer analyses means less administrative resources allocated to data analysis for the trial results and fewer delays.

**TABLE 4:** Sample allocation for the P2SD and reference methods when $\lambda_{1}=\lambda_{2}=500$, and $\mu =\mu^{*}=\Delta (1,2/3,1/3)$.

Method		Control	Arm 1	Arm 2	Arm 3	Total
**P2SD**	**Min**	45	45	45	45	180
	**Max**	120	120	120	120	330
	**Mean**	79.4±0.2	82.4±0.2	65.4±0.2	50.1±0.1	277.3±0.6
	**Percentage**	28.6%	29.7%	23.6%	18.1%	100.0%
**KST** (ϵ=0.05)	**Min**	25	25	25	25	100
	**Max**	125	125	125	125	500
	**Mean**	64.6±0.2	49.9±0.2	36.8±0.2	28.4±0.1	179.9±0.6
	**Percentage**	35.9%	27.7%	20.5%	15.8%	100.0%
**DTL** (3:2:1)	**Min**	120	40	40	40	360
	**Max**	120	120	120	120	360
	**Mean**	120	111.6±0.2	77.6±0.3	50.8±0.2	360
	**Percentage**	33.3%	31.0%	21.6%	14.1%	100.0%
**MJW‐4 stages**	**Min**	29	29	29	29	116
	**Max**	116	116	116	116	464
	**Mean**	85.2±0.2	83.7±0.2	78.4±0.2	66.2±0.2	313.5±0.7
	**Percentage**	27.2%	26.7%	25.0%	21.1%	100.0%

Table 5 shows a comparison of the P2SD method for a true response curve different from what was originally assumed. We use the Optimum Envelope (OE) designation to indicate this method was optimized for each configuration. The comparison P2SD design was optimized for the linear response when $\mu^{\left[K\right]}=\Delta$ and applied to other response curves and when $\mu^{\left[K\right]}=\Delta /2$. With a relatively small gap in performance, we find that the P2SD method is able to perform well even if the original estimate of the true μ was incorrect.

**TABLE 5:** The robustness properties of a specific P2SD method under those 10 different true μ. The specific P2SD method is designed for the assumed response, $\mu^{*}=\Delta (1,2/3,1/3)$ under #1 (linear) with $\mu^{\left[K\right]}=\Delta$. The P2SD‐OE refers to the Optimum Envelope (OE) designation to indicate the corresponding 10 different P2SD methods with each being optimized for each configuration. The results illustrate the robustness of our proposed P2SD method with respect to the pre‐specified $\mu^{*}$ value.

Setting	Method		#1 (Linear)	#2 (LFC)	#3 (Step)	#4 (Logistic)	#5 (Convex)
$\mu^{\left[K\right]}=\Delta$	**P2SD**	$E_{\mu }[(K+1)\tau_{1}+|J|\tau_{2}]$	278.0±0.8	268.4±0.8	281.3±0.8	279.0±0.8	272.1±0.8
		$P_{\mu }(\hat{K}\in L)$	0.846	0.995	0.755	0.503	0.975
		$P_{\mu }(\delta =1)$	0.881	0.902	0.876	0.932	0.884
		Score	**405**	321	453	577	334
	**P2SD‐OE**	$E_{\mu }[(K+1)\tau_{1}+|J|\tau_{2}]$	278.0±0.8	254.4±0.8	287.8±0.8	261.4±0.8	263.0±0.8
		$P_{\mu }(\hat{K}\in L)$	0.846	0.991	0.760	0.479	0.974
		$P_{\mu }(\delta =1)$	0.881	0.885	0.902	0.928	0.871
		Score	**405**	**317**	**450**	**548**	**330**
$\mu^{\left[K\right]}=\Delta /2$	**P2SD**	$E_{\mu }[(K+1)\tau_{1}+|J|\tau_{2}]$	309.4±0.8	304.2±0.8	308.9±0.8	309.2±0.8	305.3±0.8
		$P_{\mu }(\hat{K}\in L)$	0.635	0.875	0.650	0.428	0.798
		$P_{\mu }(\delta =1)$	0.308	0.276	0.305	0.397	0.275
		Score	549	424	542	652	463
	**P2SD‐OE**	$E_{\mu }[(K+1)\tau_{1}+|J|\tau_{2}]$	247.0±0.5	256.2±0.6	252.2±0.5	236.7±0.5	266.5±0.5
		$P_{\mu }(\hat{K}\in L)$	0.593	0.845	0.618	0.400	0.784
		$P_{\mu }(\delta =1)$	0.273	0.258	0.279	0.342	0.255
		Score	533	404	518	620	439

## 6 Discussion

In this paper, we address the challenge of efficiently making good decisions in the design of clinical trials to select one treatment from many. We take a pragmatic position on the primacy of prioritizing the penalized probability of events. Inspired by the Kiefer‐Weiss problem, we propose a new optimization problem which captures the relevant performance metrics, including the probability of making a good treatment selection, with financial constraints from the sample size as well regulatory constraints with the FWER. We optimize the parameters for the decision boundary, allocation of samples between stages, and treatment selection criterion using a stochastic optimization algorithm, simulated annealing.

The value of the proposed method lies in the ability to adapt to arbitrarily assigned response curves. Specifically, it has adaptive arm selection and stopping along with adaptive sample allocation in the second stage. The P2SD method also decouples the decision on the hypothesis from the treatment arm selection which improves efficiency. Researchers can incorporate prior knowledge of the mean vector in the optimization function to tune the design for their specific needs. Additionally, we show that the proposed method generalizes well to response curves other than the one it was explicitly trained on. The quantifiable performance of the proposed method compares favorably to a variety of design families both sequential and fixed. The two‐stage design also favors consistent sample sizes which benefit the teams conducting the trial. The process for making decisions on the hypothesis and treatment selection is simple to communicate with regulators and internal teams for improved transparency. Both regulators and medical researchers should benefit from the direct control over the probability of making a good decision for the treatment arm selection.

While we illustrate our main ideas under the assumptions of known common variances, the ideas can easily be adapted to more practical scenarios with unknown variances. For the test statistics $Z_{j}^{\left(k\right)}$ in (3), we can adaptively estimate the unknown common variance $\sigma^{2}$ by

(15) $$\begin{array}{l} {\hat{\sigma }}_{j}^{2}=\frac{1}{\sum_{k=0}^{K}(N_{j}-1)}\overset{K}{\underset{k=0}{\sum }}\overset{N_{j}}{\underset{i=1}{\sum }}{\left(X_{ki}-\frac{1}{N_{j}}\overset{N_{j}}{\underset{i=1}{\sum }}X_{ki}\right)}^{2}, \end{array}$$

and the corresponding updated test statistics $Z_{j}^{\left(k\right)}$ would have t‐distribution with degree of freedom of $\sum_{k=0}^{K}(N_{j}-1).$ In particular, at the end of the first stage, the degree of freedom of $Z_{j=1}^{\left(k\right)}$ is $(K+1)(\tau_{1}-1),$ as $N_{j=1}=\tau_{1}$ for all j. The t‐distribution $T_{\nu }$ with a number of degrees of freedom ν has heavy tails if ν is small, but would have similar properties as the standard Normal N(0,1) distribution if ν≥30 for most practical purposes. From the optimization viewpoint, it would be challenging to optimize the penalized objective function (14) subject to the uncertainty and randomness of ${\hat{\sigma }}_{j}^{2}$ in (15). One approach is to use the estimate ${\hat{\sigma }}_{j-1}^{2}$ for the optimal design at the beginning of the jth stage but use the estimate ${\hat{\sigma }}_{j}^{2}$ for the decision‐making at the end of the jth stage. This has been widely used in statistics, for example, Stein's two‐stage procedure [28] for unknown variance. For implementation, the mvtnorm package in R has functions for multivariate t‐distribution probabilities. When adapting this optimization to an unknown variance, we can ensure that solutions allocate sufficient samples to the first stage through the candidate generation. In this case, we could generate candidates with a lower bound on $m_{1}$. Alternatively, we could implement a constraint on $m_{1}$ such that the feasible set of solutions would be those with a minimum number of samples in the first stage for variance estimation.

One interesting research direction is to add a new arm at the interim analysis. In the case where two arms have relatively equivalent performance, a new arm that is a hybrid of those two arms would provide additional information about the response curve. In addition, it is also interesting to extend this research to non‐normal distributions. The foundation of our research is built on the Kiefer‐Weiss problem, in which the symmetry of normal distributions makes problems easier: when testing $H_{0}:\mu_{k}-\mu_{0}\leq 0$ against $H_{1}:\mu_{k}-\mu_{0}\geq \Delta ,$ the maximum expected sample size occurs at the middle point $\mu_{k}-\mu_{0}=\Delta /2$. It is useful to investigate how to extend to the case when the outcome is a binary or survival variable. In this case, it is unclear how to intuitively define a suitable middle point between two model parameters of binary or survival variables, but good approximations are provided in Huffman [29] for the single arm. These extensions are practicable for large sample sizes but it is unclear whether they are feasible for small sample sizes. We will investigate the extension of Huffman's results to the K treatment arms for binary data before adapting it to survival data. Hopefully our research brings more attention to the Kiefer‐Weiss problem formulation and opens more new research opportunities in clinical trial designs.

## 7 Reproducibility

All the code for the proposed method is available at https://github.com/phorton9/P2SD.

## Funding

Y. Mei was supported in part by NSF grant DMS‐2515158.

## Conflicts of Interest

The authors declare no conflicts of interest.

## Data Availability

Data sharing not applicable to this article as no datasets were generated or analysed during the current study.

## Appendix A Example Decision

Figure A1 shows examples of four scenarios to illustrate how decisions are made with the P2SD method. While this is non‐exhaustive, it does provide context for how the trial stops or treatments are selected. On the top left, treatment 2 is the best performing arm in the first stage for which $u_{1}<z_{1}^{\left(2\right)}<\ell_{1}$ so the trial continues to stage two. Treatment 3 is below the continuity threshold $z_{1}^{\left(2\right)}\geq z_{1}^{\left(3\right)}+h$ thus arm three is dropped. In the second stage, treatment 2 is again the maximum but $z_{2}^{\left(2\right)}<u_{2}$ thus $H_{0}$ is accepted. For the top right example, treatment 1 is the maximum with both other treatments dropped. We have $u_{1}<z_{1}^{\left(1\right)}<\ell_{1}$ so the trial continues with only treatment 1 and the control advancing to the second stage where the null hypothesis is rejected. In bottom left, the trial is stopped because the maximum treatment arm, treatment 1, is small enough that $z_{1}^{\left(1\right)}<\ell_{1}$ thus $H_{0}$ is accepted. The bottom right rejects $H_{0}$ because $z_{1}^{\left(2\right)}>u_{1}$ but the trial continues because $z_{1}^{\left(1\right)}>z_{1}^{\left(2\right)}-h$. In the second stage, treatment two is selected because $z_{2}^{\left(2\right)}>z_{2}^{\left(1\right)}$.

## Appendix B Example Event

This section presents the construction of one potential scenario for a P2SD trial design when K=3, I={1,2}, $Z_{1}^{\left[K\right]}=Z_{1}^{\left(1\right)}$, and $Z_{2}^{\left[I\right]}=Z_{2}^{\left(1\right)}$. The probability to reject $H_{0}$ in the second stage is $P_{\mu }(Z_{2}^{\left(1\right)}>u_{2},Z_{2}^{\left(1\right)}-Z_{2}^{\left(2\right)}>0,h>Z_{1}^{\left(1\right)}-Z_{1}^{\left(2\right)}>0,Z_{1}^{\left(1\right)}-Z_{1}^{\left(3\right)}>h,u_{1}\geq Z_{1}^{\left(1\right)}\geq \ell_{1})$ that can be written as the integral of the multivariate normal:

$$=\int_{\ell_{1}}^{u_{1}}\int_{h}^{\infty }\int_{0}^{h}\int_{0}^{\infty }\int_{u_{2}}^{\infty }\phi (z;\mu ,\sum )\;dz$$

where μ and ∑ are:

$$\mu =\left(\begin{array}{c} (\mu_{1}-\mu_{0})\sqrt{\frac{\left(\tau_{1}+\tau_{2}\right)}{2}}\\ (\mu_{1}-\mu_{2})\sqrt{\frac{\left(\tau_{1}+\tau_{2}\right)}{2}}\\ (\mu_{1}-\mu_{2})\sqrt{\tau_{1}/2}\\ (\mu_{1}-\mu_{3})\sqrt{\tau_{1}/2}\\ (\mu_{1}-\mu_{0})\sqrt{\tau_{1}/2} \end{array}\right),\;\sum =\left(\begin{array}{ccccc} 1 & 1/2 & \rho_{1}/2 & \rho_{1}/2 & \rho_{1}\\ 1/2 & 1 & \rho_{1} & \rho_{1}/2 & \rho_{1}/2\\ \rho_{1}/2 & \rho_{1} & 1 & 1/2 & 1/2\\ \rho_{1}/2 & \rho_{1}/2 & 1/2 & 1 & 1/2\\ \rho_{1} & \rho_{1}/2 & 1/2 & 1/2 & 1 \end{array}\right)$$

where $\rho_{1}=\sqrt{\frac{\tau_{1}}{\tau_{1}+\tau_{2}}}$. With the correlation matrix, upper and lower bounds, we can use the method from Genz and Bretz to calculate the multivariate normal random variables [26].

## Appendix C Convergence Properties

**TABLE C1:** Parameter tuning for number of loops of simulated annealing with $\lambda_{1}=\lambda_{2}=500$, and α=0.025 showing the 95% confidence interval (±1.96σ).

Num Loops	5	20	50	100	200	400	600	Dif (%)
Optim Value	410.9±6.5	406.8±4.2	405.2±1.8	404.6±1.0	404.5±0.7	404.3±0.7	404.2±0.7	1.7%
Time (sec)	16.0	71.5	114.8	228.0	457.0	913.4	1381.5	

In this section, we include a table showing the computation time and optimization value as a function of the number of inner loops for each cycle of Simulated Annealing. The optimization values correspond to Figure 2.

Table C2 confirms the convergence to a relatively consistent set of design parameters.

**TABLE C2:** Parameter tuning for number of loops of simulated annealing with $\lambda_{1}=\lambda_{2}=500$, and α=0.025 for 600 iterations.

Iteration	$u_{1}$	$\ell_{1}$	$u_{2}$	h	$m_{1}$	$m_{2}$
1	2.61	−0.90	2.49	0.53	181	162
2	2.64	−0.80	2.47	0.57	174	156
3	2.59	−0.51	2.51	0.47	186	156
4	2.66	−0.34	2.45	0.51	184	150
5	2.64	−0.37	2.47	0.55	177	148
6	2.62	−0.31	2.48	0.51	184	156
7	2.61	−0.70	2.49	0.53	176	162
8	2.65	−0.25	2.46	0.55	189	144
9	2.62	−0.79	2.48	0.54	187	156
10	2.61	−0.35	2.48	0.54	191	156

## Appendix D Response Curves

Figure D1 illustrates the variety of response curves that are used in the analysis. Each of the designs comes from [25] which are then tuned for the problem from [20] with a significant effect being one with Δ=0.2488. The dashed vertical lines indicate the values of each treatment corresponding with each response curve.

## Appendix E Notation

**TABLE E1:** Summary of notation.

Symbol	Description
K	Number of candidate treatments evaluated against a placebo
$\mu_{k}$	Mean response for treatment k, where k=0,…,K and $\mu_{0}$ is the control
$\sigma^{2}$	Variance of responses (assumed known and equal across groups)
$m_{1},m_{2}$	Total sample sizes in Stage 1 and Stage 2
$\tau_{1},\tau_{2}$	Samples per arm in Stage 1 and Stage 2
J	Set of treatment or control arms proceeding to Stage 2
$K^{*}$	Treatment arm with the highest mean response
$\hat{K}$	Selected treatment
δ	Decision on the null hypothesis
$H_{0}^{\left(k\right)}$	Null hypothesis for treatment k: $\mu_{k}-\mu_{0}\leq 0$
$H_{1}^{\left(k\right)}$	Alternative hypothesis for treatment k: $\mu_{k}-\mu_{0}>\Delta$
Δ	Clinically relevant effect size threshold
$Z_{j}^{\left(k\right)}$	Test statistic for arm k at stage j, j=1,2
$u_{1},u_{2}$	Upper decision thresholds for rejecting $H_{0}$ in Stage 1 and Stage 2
$\ell_{1}$	Lower decision threshold for accepting $H_{0}$ in Stage 1
h	Distance threshold for treatment selection
α	Family‐Wise Error Rate (FWER)
β	Global Type II error rate
L	Set of treatment arms that continue to Stage 2
